# Embracing firefly flash pattern variability with data-driven species classification

**DOI:** 10.1038/s41598-024-53671-3

**Published:** 2024-02-10

**Authors:** Owen Martin, Chantal Nguyen, Raphael Sarfati, Murad Chowdhury, Michael L. Iuzzolino, Dieu My T. Nguyen, Ryan M. Layer, Orit Peleg

**Affiliations:** 1 Department of Computer Science, University of Colorado Boulder, Boulder, CO USA; 2 BioFrontiers Institute, University of Colorado Boulder, Boulder, CO USA; 3 Santa Fe Institute, Santa Fe, NM USA; 4 Department of Civil and Environmental Engineering, Cornell University, Ithaca, NY USA; 5 Department of Physics, University of Colorado, Boulder, CO USA; 6 Department of Applied Math, University of Colorado, Boulder, CO USA; 7 Department of Ecology and Evolutionary Biology, University of Colorado, Boulder, CO USA

**Keywords:** Ecology, Zoology, Ecology

## Abstract

Many nocturnally active fireflies use precisely timed bioluminescent patterns to identify mates, making them especially vulnerable to light pollution. As urbanization continues to brighten the night sky, firefly populations are under constant stress, and close to half of the species are now threatened. Ensuring the survival of firefly biodiversity depends on a large-scale conservation effort to monitor and protect thousands of populations. While species can be identified by their flash patterns, current methods require expert measurement and manual classification and are infeasible given the number and geographic distribution of fireflies. Here we present the application of a recurrent neural network (RNN) for accurate automated firefly flash pattern classification. Using recordings from commodity cameras, we can extract flash trajectories of individuals within a swarm and classify their species with an accuracy of approximately seventy percent. In addition to its potential in population monitoring, automated classification provides the means to study firefly behavior at the population level. We employ the classifier to measure and characterize the variability within and between swarms, unlocking a new dimension of their behavior. Our method is open source, and deployment in community science applications could revolutionize our ability to monitor and understand firefly populations.

## Introduction

Nocturnal fireflies (Coleoptera: Lampyridae) have evolved a visually impressive light-based communication system under simultaneous evolutionary pressures to advertise their species, accentuate their sexual fitness, and avoid predation^1–3^. Using a luciferin-luciferase reaction within their abdomen^4^, fireflies broadcast their species identity via light pulses^1,5^. In many North American genera both sexes flash and must also encode biological sex in their signals^1,6,7^. Sympatric species, or those that share the same geographic area, must also produce distinguishable patterns to effectively communicate species identity^1,5,8^.

This unique signaling system makes fireflies particularly susceptible to human-created population threats like light pollution^9–12^. Artificial light at night (ALAN) interferes with fireflies’ perception of conspecific signals and disrupts their communication timings, curtailing flash signaling behavior and preventing successful mating^10,13^. In addition to light pollution, habitat degradation, pesticide use, water pollution, and climate change comprise some of the most serious environmental stressors causing declines in firefly populations across the globe^14^. Recent Red List assessments by the International Union for Conservation of Nature (IUCN) have identified that at least 14% of 132 firefly taxa in the United States alone are in danger of extinction^12^, but this value is a likely underestimate due to a lack of information on over half the species assessed^6,12^. Further studies and fieldwork are urgently needed to monitor changes in firefly abundances and evaluate and mitigate the threats imperiling fireflies worldwide^6,12^. Many other insect species share these environmental threats and are experiencing dramatic declines^10,15,16^. Because of their charismatic nature and popular appeal, fireflies can serve as a flagship symbol to foster public attention toward this conservation crisis.

**Figure 1:**
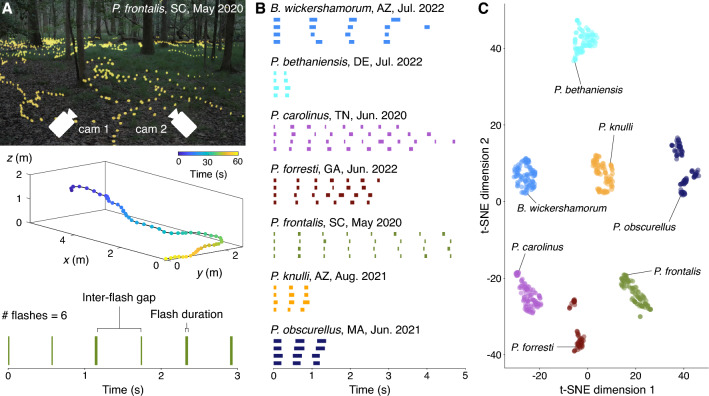
(**A**) Our standardized data collection method (top) films fireflies in their natural habitat with two cameras arranged in a stereoscopic vision configuration^22^ (photo reproduced and modified with permission from^23^). Flash streaks (center, colored by time) in the resulting videos are triangulated and concatenated into trajectories, based on proximity and velocity (bottom). Each trajectory is represented by a time series of individual flashes. (**B**) Four example five-second flash sequence time series for each of the seven species in our study, labeled with the location and date of the corresponding recording. Our recurrent neural network is used to classify flash sequence time series; time series shown were selected from the top 100 sequences per species with the highest classification probabilities following the filtration process outlined in Methods Section "Characterization". (**C**) Two-dimensional t-SNE embedding of the output of the last hidden layer of the network on the top 100 predictions, just before inference. Flash patterns are clustered by similarity, and the distinguishability of each species’ characteristic flash pattern can be detected by the colored clusters.

The major barrier to planning effective conservation efforts is the dearth of quantitative data on firefly populations^6,12,14,17^. The high-throughput population monitoring studies that provide the fundamental data for understanding population-level dynamics^18^ have not been performed for nearly all firefly species^6^. This gap is largely due to limitations of existing monitoring methodologies, which require the presence of human expert observers^6^, are often subjective^6^, and typically characterize firefly flash behavior by single, imprecise pictorial representations despite known temperature dependence and individual variability^7,8^. To address the data deficiency in current conservation efforts, we propose a scalable, automated population monitoring method that combines recent advances in stereoscopic filming, computer vision, and machine learning to classify individuals and quantify swarm-level dynamics. Our method starts with a field recording of a swarm using two consumer-grade cameras (Fig. 1A), produced following procedures described previously in^19^. From this recording, we identify individual firefly trajectories and extract time series representing their flash patterns (Fig. 1B). We then trained a recurrent neural network (RNN) on these data to accurately determine species identity from nothing more than the temporal differences in each species’ flash pattern, achieving precision and recall of approximately 0.8 and 0.6, respectively (Table 1). We additionally visualize the distinguishability of firefly flash patterns via a dimensionality reduction of the weights of the last hidden layer of the neural network, using t-distributed stochastic neighbor embedding (t-SNE)^20^. The t-SNE embedding reveals significant clustering by species (Fig. 1C). To our knowledge this is the first application of machine learning to firefly behavioral biology.

Using the most probable predictions of each species, we also provide estimates of flash pattern variability, including the first-ever quantitative characterization of *Bicellonycha wickershamorum* and *Photuris forresti*, two formerly data-deficient species which may be severely threatened^21^. Automated and data-driven methods like the one proposed here are essential to scaling ecology and conservation biology projects^17^. This work enables accurate identification and classification of firefly species in order to ensure their protection and long-term survival.

## Results

Using our precise, high-throughput data acquisition method (Fig. 1A and Methods Section "Acquisition of flash sequence data"), we recorded nine firefly swarms during the summer months of 2020, 2021, and 2022 that comprise seven North American firefly species: *Bicellonycha wickershamorum*, *Photuris bethaniensis*, *Photinus carolinus*, *Photuris forresti*, *Photuris frontalis*, *Photinus knulli*, and *Photinus obscurellus*. This resulted in a total of 65,389 flash pattern time series after cleaning (see Methods Section "Acquisition of flash sequence data" and Ref.^24^ for the original dataset). These data are a dramatic expansion from previously published results, which were primarily a single characteristic pattern per species. This large dataset enables us to leverage machine learning for species classification and characterization of intra-swarm variability.

### Model performance

We trained a recurrent neural network (RNN), a type of neural network ideal for classifying time series and periodic signals, on the dataset in Ref.^24^ to predict the species that produced each sequence (see Methods Section "Neural network architecture"). The accuracy of these predictions was compared to those obtained from several alternate classifiers utilizing signal processing metrics (Table 1 and Methods Section "Signal processing algorithms"). We present ensemble results evaluating the model’s performance on the test set for each method in Table 1. On the entire dataset, the RNN achieves the best performance in both categories, with an increase of approximately 6% and 17% in weighted precision and recall, respectively, over the second best classifier, and an overall accuracy of 69%.

**Figure 2:**
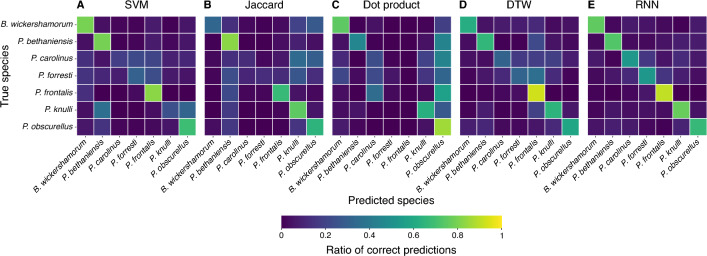
Confusion matrices for each classification method. (**A**–**E**) Ratios of true positives and false negatives along the horizontal, true positives and false positives along the vertical. Each square in the diagonal represents the recall for the class, and brighter colors indicate higher values as shown in the colorbar. A perfect classifier would consist of yellow squares at each diagonal position and dark purple off the diagonal.

We first attempt to utilize published, pictorially represented flash patterns (see Methods Section "Literature references") to classify sequences with signal processing methods. We constructed three different classifiers that use the Jaccard index, dot product, and dynamic time warping (see Methods Section "Signal processing methods") to compare our time series data to the literature references. We additionally constructed a support vector machine (SVM) that classified the time series data from the three flash pattern parameters (flash duration, inter-flash gap, and the number of flashes) (Fig. 1A, bottom). These literature-based classifiers perform poorly: while dynamic time warping achieves the highest precision with 0.73, it achieves very low recall of 0.12; dot product achieves the best recall of the literature-based classifiers, at 0.41 (Table 1).

We attribute the poor performance of the literature reference methods to the ineffectiveness of a single reference sequence per species at capturing the intra-species variability inherent to a large dataset. To address this, we create “population reference” sequences by aggregating sequences of each species in our dataset, aimed at capturing the intra-species variability while preserving species-specific characteristics of the flash signal. We split the data into 80% for training and 20% for testing: we generate population references from the training set, and classify the remaining test data. The performance of all four classifiers improves slightly by using population references, with dynamic time warping achieving the highest precision and recall of 0.77 and 0.47, respectively (see Table 1). Additionally, we note the literature are incomplete with regard to endangered species such as *B. wickershamorum*, one of the species most represented in our dataset.

**Table 1:** Summary of signal classification methods and their weighted average performance on the firefly flash pattern dataset.

Metric	Precision		Recall	
	Literature	Population reference	Literature	Population reference
SVM	0.67	$0.75 \pm 0.0046$	0.10	$0.38 \pm 0.015$
Jaccard index	0.57	$0.79 \pm 0.0056$	0.27	$0.29 \pm 0.021$
Dot product	0.57	$0.53 \pm 0.017$	0.41	$0.31 \pm 0.027$
Dynamic time warping	0.73	$0.77 \pm 0.0040$	0.12	$0.47 \pm 0.0052$
RNN	$0.83 \pm 0.0014$		$0.64 \pm 0.0020$	

± values indicate standard error of the mean for each statistic.

In Fig. 2 we show confusion matrices for each ensemble to further illustrate the per-class capabilities and shortcomings of each method. While the dynamic time warping and dot product classification methods occasionally produce a high scoring class (see Methods Fig. 6F), the RNN results reveal a balanced, highly accurate classifier regardless of species, and as such is our recommendation for the algorithm of choice moving forward in this space. Per-species precision and recall metrics can be found in Methods Fig. 6.

### Artificial sympatry

While the species in our dataset generally do not overlap in space and time, we recognize that there can be sympatric species that share the same breeding grounds, leading to the potential for recordings that contain flashes from multiple species. However, there are challenges in obtaining experimental data with mixed recordings of firefly species exacerbated by the scarcity of isolated species in nature, the difficulty in creating controlled mixtures, and the absence of accurate data on species numbers in these recordings. Therefore, we construct an experiment to test the ability of the model to distinguish between two species in a hypothetical sympatric swarm, provided that the model has been trained on data from each of these species recorded in isolation.

First, a single recording (i.e. one night of data) for five — *B. wickershamorum*, *P. carolinus*, *P. frontalis*, *P. knulli*, and *P. obscurellus* — of the seven species in our dataset is held out from training the model (Methods Section "Sympatric species experiments"). The remaining two species only contain data from 2 recordings, and therefore are not included in this experiment due to a lack of sufficient data. Then, the model is trained on the remaining time series, with the data preprocessed as discussed in Methods Section "Data preprocessing". Mixed-species datasets are constructed from 400 total sequences obtained from varying proportions of two species, ranging from 0.5% of the minority species and 99.5% of the majority species (i.e. two time series from the minority class and 398 time series of the majority class), to equal proportions of each (200 time series each).

**Figure 3:**
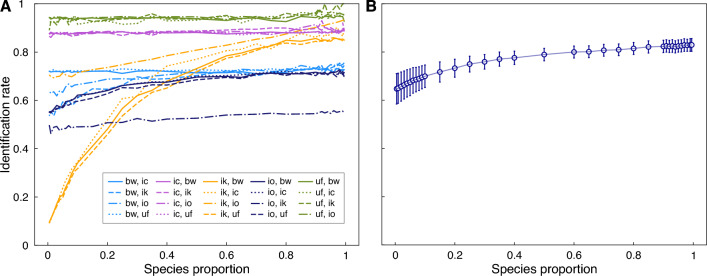
Experiments with artificial sympatric swarms reveal that the model can identify both species. (**A**) We constructed test datasets consisting of trajectories from a pair of species combined in varying proportions. For each pair of species, the identification rate is shown as a function of the species proportion for the colored species. Each colored line represents a species in varying density with another species. Legend key: bw: *B. wickershamorum*; ic: *P. carolinus*; ik: *P. knulli*; io: *P. obscurellus*; uf: *P. frontalis*. (**B**) The identification rate aggregated across all sympatric swarms decreases as a function of species proportion. On average, when two species are combined in equal amounts, the neural network classifies each species with an accuracy of close to 80%. Even with as few as two trajectories present in a sympatric swarm, the model will still detect the presence of that species more than half the time across all pairs.

We perform 500 iterations, wherein we randomly sample one of these test datasets from the held-out data and evaluate the average true response rate for both species. We repeat this for all possible pairs of species (20 pairs total). The identification rate as a function of species proportion is shown in Fig. 3 for each pair (panel A), as well as the aggregate result (panel B). We observe that some species are more distinguishable, with *P. frontalis* and *P. carolinus* achieving a high identification rate even when represented in very low proportions. Meanwhile, *P. knulli* is more readily confused with other species, achieving an identification rate of 50% only when present in at least a 20% proportion if together with any other species except *P. obscurellus*. *P. obscurellus* and *P. knulli* are frequently confused for one another – possibly as these species have similar numbers of flashes in their flash patterns (Fig. 1B) – with *P. obscurellus* barely achieving 50% identification even when it is present in a high proportion with *P. knulli*. Altogether, the aggregate results show that when two species are present in equal proportion, the model can differentiate between the two with nearly 80% accuracy. This indicates that the classifier has the potential to be successful in differentiating sympatric species, which we expect to be a likely future application of the model.

### Flash characteristics

By combining our extensive data set and effective RNN classification system, we provide deeper insights into firefly behavior with a data-driven characterization of the signaling patterns of firefly populations. For each swarm recording, we aggregate the one hundred most confidently classified flash patterns (see Methods Section "Characterization") to produce empirical distributions for several trajectory-level statistics. These are the number of flashes in an individual’s flash pattern; the flash duration, or the amount of time for which a flash is detected; and the inter-flash gap, defined as the amount of time from the end of a flash to the start of another flash (i.e. the dark period within a flash pattern).

We note that we measure inter-flash gap, defined as the duration of the dark (quiet) period between flashes, rather than the more commonly used inter-flash interval, which corresponds to the time period measured from the start of one flash to the start of the subsequent flash and thus includes one flash duration. We measure the gap rather than the inter-flash interval in order to examine variability in flash duration and pauses independently, whereas examining flash duration and inter-flash interval would not easily allow us to separate whether variation stems from the duration spent flashing or the time spent waiting between flashes. Additionally, the inter-flash gap as a quantity is only well-defined for flash patterns that exhibit two or more flashes in relatively quick succession, because measuring this quantity for an individual trajectory requires that flashes are close enough in space and time to be concatenated (see Methods Section "Acquisition of flash sequence data"). Our methodology cannot be well applied to species that flash only once in a pattern with long dark periods between flashes (see Discussion Section "Data acquisition methodology").

**Figure 4:**
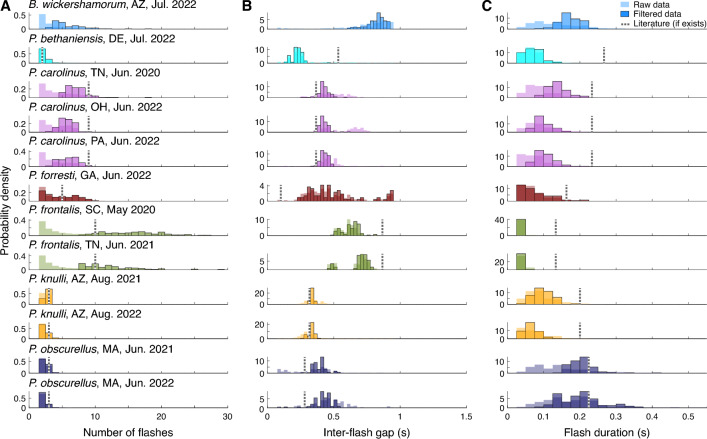
Data-driven characterization of flash patterns for each filmed population, encompassing 7 different species. *P. carolinus* and *P. frontalis* data were each collected from two populations filmed in different locations and years, each of which is separately characterized here. For each population, the 100 sequences with the highest classification probabilities are used to characterize flash signals. This represents a data filtering procedure that extracts the most salient properties of each population’s flash behavior while retaining inherent intraspecies behavioral variability. Probability distributions of the (**A**) number of flashes, (**B**) inter-flash gap in seconds, and (**C**) flash duration in seconds are shown for each species, normalized to sum to 1 under the interval, for both the raw (transparent bars) and filtered (opaque bars) data. The corresponding values obtained from the literature references (see Methods Section "Literature references"), if they exist, are shown as dashed lines.

Quantitatively derived distributions for these characteristic statistics are shown in Fig. 4. These distributions represent the first known quantification of firefly behavioral variability from data. Table 2 compares the mean and standard deviation of flash parameters from our data with the published literature values, where available.

Encouragingly, previously published values^2,6,19^ for the number of flashes in each individual flash pattern were statistically indistinguishable from our distributions (Fig. 4A and Table 2). However, half of the published inter-flash gaps and all the published flash durations differed significantly from our distributions. These differences may be due to improved measurement accuracy (see below) or could represent behavioral differences between swarms, perhaps caused by a changing climate or other environmental factors.

These data-driven distributions have several advantages over manual timekeeping. Firstly, representing a species’ flash-based communication as distributions of signal parameters rather than a single pattern allows us to explore the extent of the temporal variability present in a population or across populations. We observe, for example, high variability in the number of flashes emitted by *P. frontalis*, while the flash patterns of *P. knulli* exhibit very low variability in the number of flashes (Fig. 4A). Meanwhile, both of these species display relatively tight distributions of inter-flash gap (Fig. 4B), suggesting that these species require precise timing of flashes for communication.

Our results reveal that the flash length duration distributions for five species are shifted considerably toward shorter flash durations than the published literature results (see Fig. 4C). This discrepancy may potentially be a consequence of the lower temporal resolution inherent in manual timekeeping due to system lag. However, this may also be a limitation of our study (see Discussion Section "Data acquisition methodology"), due to the light sensitivity of the GoPro cameras employed for data acquisition.

Overall, the filtering process that produces data-driven characterizations of flash patterns in Fig. 4 preserves the most salient characteristics of each species’ signaling behavior while also illustrating the intraspecies behavioral variability. This variability may be substantial in only some of the 3 parameters: for example, *P. obscurellus* demonstrates low variability in the number of flashes but the largest variability in flash duration out of the 7 species studied. In comparison, the flash durations of *P. frontalis* sequences are all extremely similar, but the number of flashes in a trajectory can vary greatly.

We note that these characterizations only represent the locations and conditions where the underlying data were recorded; these probability distributions may be different for data taken in different habitats, at different temperatures, or at different times (see Sect. "Temperature variation"). By deploying our data acquisition procedure to more sites, we will be able to better characterize the rich extent of a firefly species’ communication.

**Table 2:** Mean and standard deviation of the number of flashes, inter-flash gap, and flash duration from the filtered data for each filmed population, along with the corresponding literature values (where available) extracted via methodology shown in Fig. 5.

Species	Number of flashes		Inter-flash gap (s)		Flash duration (s)	
	Data	Literature	Data	Literature	Data	Literature
*B. wickershamorum*, AZ, Jul. 2022	$5.9 \pm 2.5$	n/a	$0.82 \pm 0.07$	n/a	$0.17 \pm 0.03$	n/a
*P. bethaniensis*, DE, Jul. 2022	$2.3 \pm 0.6$	2	$0.22 \pm 0.04$	0.53	$0.07 \pm 0.03$	0.27
*P. carolinus*, TN, Jun. 2020	$7.2 \pm 1.9$	9	$0.42 \pm 0.04$	0.37	$0.14 \pm 0.03$	0.23
*P. carolinus*, OH, Jun. 2022	$5.7 \pm 1.1$	9	$0.42 \pm 0.04$	0.37	$0.10 \pm 0.02$	0.23
*P. carolinus*, PA, Jun. 2022	$5.9 \pm 1.4$	9	$0.43 \pm 0.03$	0.37	$0.11 \pm 0.03$	0.23
*P. forresti*, GA, Jun. 2022	$4.7 \pm 2.3$	5	$0.51 \pm 0.20$	0.10	$0.08 \pm 0.04$	0.16
*P. frontalis*, SC, May 2020	$15.7 \pm 4.3$	10	$0.63 \pm 0.07$	0.87	$0.04 \pm 0.003$	0.13
*P. frontalis*, TN, Jun. 2021	$12.2 \pm 4.1$	10	$0.69 \pm 0.09$	0.87	$0.04 \pm 0.005$	0.13
*P. knulli*, AZ, Aug. 2021	$2.7 \pm 0.4$	3	$0.34 \pm 0.01$	0.32	$0.10 \pm 0.03$	0.20
*P. knulli*, AZ, Aug. 2022	$2.4 \pm 0.6$	3	$0.33 \pm 0.02$	0.32	$0.06 \pm 0.02$	0.20
*P. obscurellus*, MA, Jun. 2021	$2.4 \pm 0.6$	3	$0.39 \pm 0.05$	0.28	$0.21 \pm 0.05$	0.22
*P. obscurellus*, MA, Jun. 2022	$2.2 \pm 0.4$	3	$0.42 \pm 0.07$	0.28	$0.23 \pm 0.12$	0.22

## Discussion

In this paper, we present the first use of artificial intelligence as a tool to empower firefly conservation by automatically classifying firefly species from their flash patterns. By integrating an inexpensive data-gathering procedure with this method, we now have the core components of an automated swarm monitoring system that aligns with biodiversity outcomes suggested in^17^ and^21^. There are 171 known firefly species in North America^6^, many of which are endangered and/or lack quantitative data on their flash behavior; these knowledge gaps significantly limit our ability to comprehend and respond to environmental pressures. Our work takes substantial steps toward filling these gaps by presenting a quantitative framework for characterizing behavioral variability, including in two species that lack an existing description of their behavior in the literature. In addition, we show that our classification method is applicable to groups of fireflies containing at least two species, a likely scenario in future acquired data. Ultimately, our results have significant ecological and conservation applications for *Lampyridae* insects; however, as we indicate below, there remain some limitations that should be responsibly addressed in future work in order to leverage our model at a large scale.

### Limitations

#### Sympatric firefly species

While this work can be immediately useful to the firefly conservation community in areas with known populations of specific species, there are some crucial limitations. First, adding new species to the dataset requires clean, labeled data obtained from video recordings of that species in isolation from any other confounding light signals. For example, some of the recordings in the previously published dataset^24^ potentially contain flash signals from multiple species in the same location. It would only be possible for these sympatric species to be successfully classified once clean data for each species in isolation has been incorporated into the training set. Should this be the case, the model could be used to classify signals between sympatric species, as we show in Fig. 3. However, our current model has been trained on the flash patterns from seven species that primarily occur in their own space and time during their nightly flashing performances, so we can only speculate with simulation whether this kind of sympatric differentiation is possible on the data at hand. Despite these limitations, the results we showed in Results Section "Artificial sympatry" enable us to have confidence in the model’s classification decisions regarding presence and absence data for the species in the dataset when they appear in recordings alongside other sympatric species. We also note that character displacement has resulted in increased differentiation of sequences of sympatric species^8^ and thus sympatric data may be easier to classify. As we continue to train and evaluate the model in the future, adding new populations of existing species and updating the model’s knowledge should not be a problem.

#### Temperature variation

Using temperature as a feature in classification was not attempted for several reasons, even though it is potentially informative. First, it can be seen as a proxy for location, and since our species geographically isolated from each other, it could result in the model learning locations more than timeseries, which we tried to avoid. Second, temperature measurements were not obtained for all recordings used in our dataset^24^. However, temperature can play an important role in firefly flash behavior. Past work has generally standardized each species’ flash patterns (at a particular temperature, usually 20 degrees Celsius^8^, and across locations^7^), resulting in single, discrete measurements for the relevant statistics that are used as references for species identification in tandem with physical observation of captured specimens. We know from our own field observations, outside consultation, and the literature that for some species, firefly behavior varies dramatically with temperature^25,26^. Our work here attempts to establish a new norm for representing firefly characteristics by accounting for the variability intrinsic within species and exacerbated by temperature. We show distributions for the flash pattern characteristics in Fig. 4, including representations for geographically distinct populations of the same species, but this is not a perfect encapsulation of the differences that can occur due to temperature. We include in the data files in our Github repository (see Section "Data availability") a column for the known temperatures so that this can be explored in future work, and note that ideal data gathering would always include temperature measurements throughout the duration of future recordings.

#### Data acquisition methodology

Our data acquisition procedure relies on a calibration-free stereoscopic reconstruction that is not without error^24^. We employed GoPro cameras for data acquisition, which are highly portable and achieve good temporal resolution (30 fps) for capturing flash behavior. However, we also observe that recorded flash durations can appear generally shorter than those measured through other techniques, possibly due to frame rate, as well as the cameras’ limited light sensitivity, which might trim the beginning and ending of a flash and hence decrease its effective duration by one or two frames. This effect can be seen in Fig. 4C. In addition, a given trajectory might not capture the entire flash pattern of an individual, due to overlapping signals from nearby fireflies, prolonged dark periods between series of successive flashes, and possible visual obstruction of flashes from the environment. Hence, it can be difficult to estimate the total count of flashes in a firefly’s signal, and the dark period between flash phrases is fully lost in most trajectories. Combining our method with continued observations by human experts for the most complete characterization of any given species’ flashing behavior may be the optimal approach in the short term, especially in species known to have dark periods that exceed one second in length between flash phrases, or species that produce a single flash, since these species are difficult to trajectorize. The advancements presented in this paper will indisputably accelerate this process and should only improve in effectiveness over time.

#### Model methodology

We estimate that at least one hundred trajectories per species are required before the model can robustly distinguish their patterns, which typically requires a few days of filming. The model classification relies only on the temporal information present in each sequence, and ignores metadata such as temperature, location, and spatial components of the flash pattern. This is intentional to allow this work to serve as a proof of concept that classification via temporal variation is possible, and to avoid confounding factors that may bias the model. In particular, given the limited size of our dataset, we expect that including the temperature and location data as features for the model would only serve as to teach the model to differentiate by those factors, and thus result in a model that would not generalize well to unseen data. Long-term, it may become prudent for these factors to be included, especially as we gather more consistent year-over-year data for each species.

### Advancing firefly conservation

The affordability and portability of the filming system makes it ideal for deployment by researchers and citizen science volunteers across the continent, and beyond. Our automated trajectory detection method enables measurement of the flash durations and inter-flash gaps (Fig 4, Table 2) of firefly signals, aggregated from thousands of precise observations of the same species across multiple nights of filming.

Our flash sequence characterization of *B. wickershamorum* and *P. forresti* represent the first known quantitative description of the flash pattern for these vulnerable species^6^. Additionally, all the characterizations as presented here, temperature-based or otherwise, can immediately be used to inform field scientists about the range of behavior they should expect to see in the flash parameters of these species. We also may be able to deploy this model on other flash sequence recordings in new areas and determine whether species that are already in the dataset are present in those new areas. Perhaps most impactfully, species living in known locations can be automatically monitored for behavioral or population density changes; this is especially valuable for threatened species (*B. wickershamorum*, *P. bethaniensis*, *P. forresti*, and *P. knulli*). It is also possible to detect changes in behavior or activity at a known site by comparing new observations to historical data. For example, despite the variability in inter-flash gaps, our model produced accurate classifications for *P. frontalis* swarms collected at different sites, by different individuals, and under different conditions (Supplementary Section 1).

Previous studies have examined the effect of environmental factors such as ALAN on firefly signaling by quantifying the reduction in flash rate or the number of flashing individuals^10^. Our method of generating data-driven characterizations will enable a more acute investigation of how ALAN and other factors affect firefly flash pattern parameters down to an individual level. In addition, we can investigate whether responses to environmental factors are encoded in behavioral variability: it is unknown whether the variability in flash behavior changes with light pollution and the resulting implications for firefly signal processing and mating success. Going forward, all species should be targeted for future deployments to establish their flash dynamics baseline and continue with periodic monitoring, with priority given to data-deficient species and species at current conservation risk. Long-term, it will be possible to differentiate between signals from footage of swarms that contain multiple species.

As the capabilities increase, the classifier can be integrated into community-facing applications like iNaturalist^27^, which in turn will enable faster data-training-classification loops and a widespread expansion of the model’s reach. Integration within community science is a vital component of the next steps: as discussed in^28^, community monitoring programs have the potential to raise the public’s awareness and understanding about firefly endangerment and promote successful integration of policy and practice moving forwards. Our standardized, user-friendly data acquisition procedure can be adopted by volunteers and researchers to grow the dataset, with an eye toward recording flash sequences from data-deficient species. We intend for this work to illuminate the way forward for increased data-gathering, population monitoring, and analysis efforts surrounding *Lampyridae* insects. Our software is open-source and the dataset is freely available, so anyone who shares our goal of empowering firefly conservation efforts is welcome to contribute.

## Methods

### Acquisition of flash sequence data

To extract flash sequence data, we perform 3D reconstruction of firefly swarms based on stereoscopic video recordings. Recordings were conducted at specific locations across the country where certain species were known to emerge^24^. Field researchers placed two spherical (360) GoPro Max cameras at known distances from each other on a level surface (Fig. 1A). Recordings started at dusk when the first flashes were seen, and filmographers performed a simultaneous catch-and-release identification process to acquire ground-truth labels from visual inspection of the individuals present in the swarm. All recordings are made at a frame rate of 30 frames per second. The movies were subsequently processed as described in a previous work^22,24^ to extract the 3D locations of flash occurrences. From these locations, we apply a simple distance-based linkage method to concatenate flashes into streaks and streaks into trajectories. We consider flashes at consecutive timesteps within a small radius to be part of the same streak; streaks occurring within both 1s and 1m of each other are assumed to come from the same individual and placed in a set of transitively connected streaks called a trajectory. To eliminate noise effects from the trajectory extraction, we threshold the trajectories to eliminate those that only contain one flash. The dataset^24^ includes ten total species before the application of the thresholding process. Following the thresholding, we also remove any species from the dataset that have fewer than one hundred total trajectories, leaving us with seven species total. Finally, from the trajectories, we extract a binary time sequence by considering the time coordinates of flashes, i.e. a sequence of ones and zeroes where ones represent flashing and zeroes represent interflash gaps. Each element (or bit) of the time series represents a single frame of a video, such that 30 bits represents 1 full second of recording. We further clean the dataset by recognizing that any interflash gaps 1 or 2 bits in length (less than 0.07s) are likely caused by an error in the tracking or trajectorization process, or the firefly briefly being obscured by brush as it moves. These short gaps are replaced by ones to connect the interrupted flash.

This process enables the capture of individual behavior from simple footage of firefly swarms of any species, provided individuals of that species flash frequently enough to meet the threshold standards of the trajectory generation. Our data acquisition procedure highlights the presence of intraspecies behavioral variability, and characterizes this variability by representing flash patterns as distributions (Fig. 4A–C).

The result of this process is 124,503 flash trajectories from the seven species before thresholding, and 65,389 after those with only one flash have been removed. More than half of these are *P. carolinus* sequences - the majority class. About 1 percent of these are *P. forresti* and *P. bethaniensis* sequences - the minority classes. The rest of the classes range between 4 and 14 percent of the total distribution. The dataset comprises binary sequences of between 6 and 1366 bits (0.2s to 45.5s) in duration, each labeled with the corresponding firefly class.

### Modeling

We implemented a bespoke neural network architecture with PyTorch to solve our classification problem. For the curious, technical details about the implementation and data-wrangling practices follow. Additionally, all code is open source and available as mentioned in Section "Data availability".

#### Neural network architecture

RNNs are a class of neural networks suitable for sequence learning tasks. They are characterized by feedback connectivity and the consequent ability to encode long-range temporal dependencies, such as those intrinsic to firefly flash patterns. The defining computational step of an RNN is the hidden state update, which is a function of the input at the current timestep, $x^{(t)}$, and the hidden state at the previous timestep, $h^{(t-1)}$:

1 $$\begin{aligned} h^{(t)} = f(W_{hh} h^{(t-1)} + W_{xh} x^{(t)} + b), \end{aligned}$$

where *f* is a non-linear activation function, such as hyperbolic tangent, $W_{hh}$ and $W_{xh}$ are weight matrices that map hidden-to-hidden (i.e. the feedback connectivity) and input-to-hidden, respectively, and *b* is a bias term.

Importantly, Eq. (1) enables a recurrent neural network to ingest variable length input sequences, as the update rule can be applied recurrently, which is suitable for firefly flash sequences that are of variable temporal duration. However, if the input duration is sufficiently large–as is the case with some of flash pattern sequences–vanishing gradients will arise when computing weight updates via backpropagation through time (BPTT)^29^ due to the non-linearity in *f*, ultimately prohibiting learning.

To address this issue, we leverage an extension to RNNs–gated recurrent units (GRUs)–that introduces gating mechanisms that regulate which information is stored to and retrieved from the hidden state^30^. These gating mechanisms enable the model to more effectively regulate the temporal context encoded in the hidden state and enables the encoding of longer-range temporal dependencies. Additionally, GRU RNNs are computationally more efficient than other kinds of RNNs like long short-term memory networks (LSTMs), and use fewer parameters, which was a consideration due to our plans for eventual downstream application of the model in real-time population monitoring. Consequently, we implement the model in PyTorch^31^ as a 2-layer GRU with 128-dimension hidden layers, no dropout, and LeakyReLU activation layers with a negative slope of 0.1.

#### Data preprocessing

To evaluate our model’s predictive ability on unseen data, we perform 60-fold stratified cross validation to ensure that each sequence in the dataset is used at least once in training and at least once in testing, but never simultaneously. Each fold divides the data into ninety percent training, and ten percent testing, preserving the same class ratios as the original dataset. Due to the severe class imbalance (e.g. some species only comprise 1% of the dataset, whereas others comprise close to 50% of the dataset), we perform random undersampling on the training set of each fold to equalize the class count in the training and validation sets for each fold. This takes the form of a secondary k-fold cross validation procedure to sample from each class until classes are equalized. All the remaining data are used for testing. The reported results are thus the ensemble precision, recall, and accuracy of each model on its respective test set of approximately 30,000 sequences, averaged over the 60 model folds. The ground truth targets are species names; we performed label encoding to transform the targets into machine-readable integers representing each class.

#### Training and evaluation

We trained the model with the Adam optimizer^32^ and evaluated performance via cross-entropy loss. During training, we set an early stopping callback that monitored the validation loss with a patience of 50 epochs to prevent overfitting on the training set. Additionally, to alleviate exploding gradients, we applied a gradient clipping of 0.1 to the gradient norms following the procedure recommended in Ref.^33^. We conducted a hyperparameter sweep over the batch size and learning rate, testing all combinations of batch size $\in \{8,16,32\}$ and learning rate $\in \{10^{-3},10^{-4},10^{-5}\}$. We selected the combination that had the highest validation set accuracy on a four-species subset of the data, which resulted in the choice of a batch size of 8 and a learning rate of $10^{-5}$. No data augmentation was applied during training.

We evaluate the performance of the RNN, along with the signal processing methods described in the following section, on the test data by examining the receiver-operating characteristic (ROC) curves for each species (Fig. 6A–E). Per-species precision and recall are tabulated in Fig. 6F.

#### Sympatric species experiments

To explore the capabilities of the model when faced with sympatric swarms, we first gave the model a different training regimen. All of the data except for sequences from five days – one day each for *B. wickershamorum*, *P. carolinus*, *P. frontalis*, *P. knulli*, and *P. obscurellus* – serve as the training and validation set, and sequences from the five held-out days enter the test set. The five held out days and their codes as referenced in^24^ are as listed in Table 3. Holding out single days like this ensures that a) the sequences being tested do not occur in the test set and b) the model can identify new sequences on a new day for a species it has already seen before.

**Table 3:** Metadata of held-out sequences for sympatry experiments.

Day	Species	Dataset code
May 20, 2020	*P. frontalis*	s0524uf
June 13, 2020	*P. carolinus*	s0613ic
June 2, 2021	*P. obscurellus*	s1602io
August 9, 2021	*P. knulli*	s1809ik
June 24, 2022	*B. wickershamorum*	s2624bw

We note that *P. bethaniensis* and *P. forresti* are excluded from these experiments. This is because for both of these species, holding out one day of data would reduce the total number of sequences in the training set to below one hundred, which is against our recommendation for sufficiency. However, these species remain in the training set, as none of their dates are held out, and thus the model can still predict them during these sympatry experiments.

The goal of these experiments is to vary the different densities of each possible pair of species to test whether the model as trained can capture the presence of each species. This tests whether the model is applicable in future hypothetical scenarios where more than one species may be present in a recording, but each of the species present are already part of the training set in some form. For each experiment, we generated a test set of 400 sequences comprising two species from the holdout days, mixed together at a particular density ratio to create an articifial instance of sympatry. This means that for each iteration of the experiment, the number of sequences for each species ranged from two to 398. We ran 500 iterations of each density ratio for each pair, where each iteration was randomly sampled from the set of sequences corresponding with the held out date. We recorded the true positive rate for each class at each density reported by the model. The results are shown in Fig 3.

### Signal processing methods

For the purposes of comparison with the RNN, we implemented four alternative classifiers which use standard signal processing algorithms to compare our dataset against ground truth references for each species. We implement these classifiers using two types of ground truth references: “literature references”, which use flash patterns as previously published in the literature, and “population references”, which are generated by aggregating sequences in our own dataset.

#### Literature references

“Characteristic” flash patterns for six out of the seven species analyzed in this paper, excluding *B. wickershamorum*, have been previously recorded and published in the literature. These recorded flash patterns hence served as the primary reference for researchers in identifying signals observed in the field. These reference flash patterns are typically reported pictorially; thus, we convert images to binary-valued time series by computing the relative sizes of flashes and gaps, in pixels. We determine the pixel-to-second conversion to then convert the sequence to a 30 frames per second time series, matching the sampling frequency of our data. We have quantified these variables from published works to underscore the prevalent tendency toward qualitative approximations over quantitative analyses. Flash signals are commonly documented in scholarly articles and monographs through visual representations, frequently drawing from multiple, and occasionally ambiguous, primary and secondary information sources for individual species.

These six reference time series then form the ground-truth comparisons against which our dataset is compared, using the four signal processing techniques described below in Methods Section Signal processing methods. We omit *B. wickershamorum* as there is currently no published reference pattern.

#### Population references

We also generate “population references” by aggregating sequences in our own dataset. For each species, we first perform an 80:20 train:test split, similar to the preprocessing procedure performed for the RNN (see above in Methods Section"Data preprocessing"). The population references are obtained by averaging the sequences in each training set. The remaining test data is then classified using the signal processing algorithms described below in Methods Section "Signal processing algorithms". As with the RNN, we perform $N = 100$ iterations and take the ensemble average of the performance across all iterations.

#### Signal processing algorithms

##### Jaccard index

 The Jaccard index compares the similarity between two sets by taking the ratio of the size of the intersection of the sets with the size of the union^34^ and has found broad application, for example in genomics^35^. For two binary-valued sequences $(a_m)_{m=1}^{M}$ and $(b_n)_{n=1}^N$ of lengths *M* and *N*, respectively, with $a_m, b_n \in \{0,1\}$ for all *m* and *n*, we define the size of the intersection as $\sum _{i=1}^{\text {min}(M,N)} a_i b_i$, the number of ‘on’ (flashing) bits that occur simultaneously in both sequences. We define the union as $\sum _{m=1}^M a_m + \sum _{n=1}^N b_n$, the number of on bits for both sequences combined. The Jaccard index can also be evaluated in the same manner for two sequences that are binary-valued. Generally speaking, the intersection can also be thought of as the dot product between the two sequences. To classify a sequence using the Jaccard index, the Jaccard index between a sequence and each species reference is computed, and the softmax of the vector of Jaccard index values is computed to determine a probability of the sequence being from each species. The predicted species is then the argument maximum (arg max) of the softmax vector.

##### Dot product

 The dot product between two sequences is given by the sum of the product of the sequences, i.e. $\sum _{i=1}^{\text {min}(M,N)} a_i b_i$ for two sequences $(a_m)_{m=1}^{M}$ and $(b_n)_{n=1}^N$ of lengths *M* and *N*, respectively. Sequences are then classified by taking the arg max of the softmax of the dot product with each reference.

##### Dynamic time warping

 Dynamic time warping (DTW) is an algorithm that computes the distance between two time series by locally stretching or compressing the sequences to optimize the match. DTW is useful for comparing time series that are qualitatively similar but vary locally in speed and has found significant application in speech recognition^36–39^. We implement DTW in MATLAB^40^ to compute the distance between sequences and species references. Similarly to the other metrics, the predicted species is taken to be the arg max of the softmax of the distances, which is a vector of probabilities that maps to the probability of each label.

##### SVM

 Each flash sequence can be parametrized by 3 values (Fig. 1A): the number of flashes in the sequence, the average duration of each flash, and the average time between flashes (inter-flash gap). We perform support vector machine (SVM) classification in this 3-dimensional space, using a radial basis kernel function.

**Figure 5:**
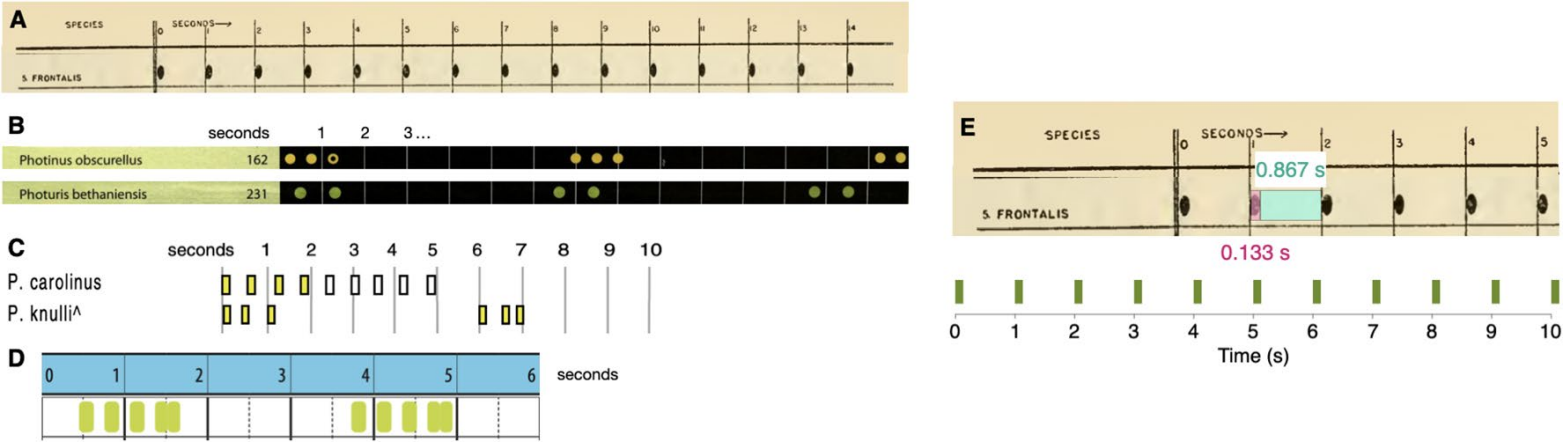
Reference sequences for the firefly species examined in this paper, as previously published in the literature, with the exception of *B. wickershamorum*. (**A**) Reference pattern for *P. frontalis* from Barber, 1951^41^. (**B**) Reference patterns for *P. obscurellus* and *P. bethaniensis* from Faust, 2017^7^. (**C**) Reference patterns for *P. carolinus* and *P. knulli* from Stanger-Hall and Lloyd, 2015^8^. (** D**) Reference pattern for *P. forresti* from Fallon et al., 2022^6^. (**E**) Illustration of extracting *P. frontalis* sequence from literature pattern (top) and converting to time series (bottom).

**Figure 6:**
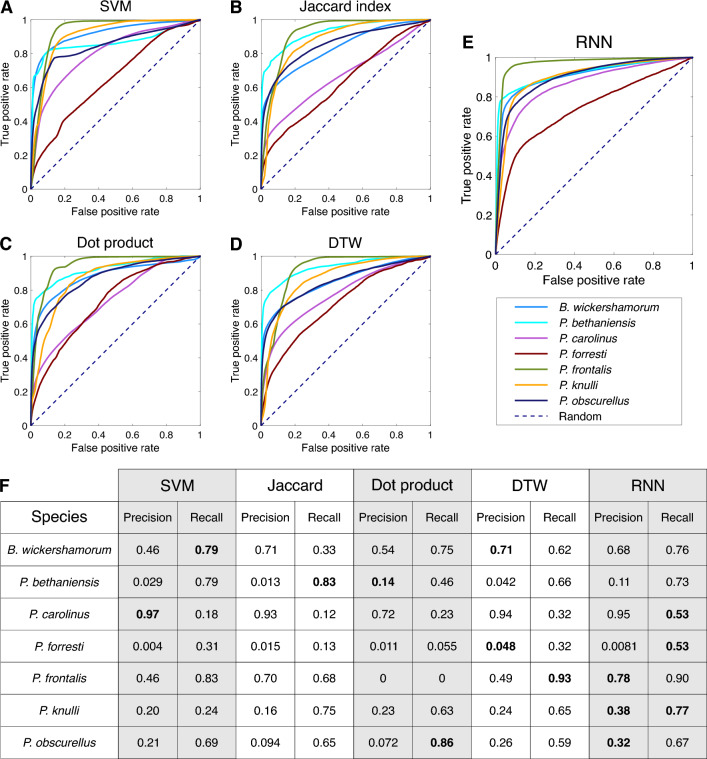
Per-species ensemble of classification results. (**A**–**E**) Receiver operating characteristic (ROC) curves representing the true positive rate (TPR) as a function of the false positive rate (FPR) across all model thresholds of classification, labeled by method. All non-RNN classification methods are conducted using population references. (**F**) Table of per-species precision and recall across all surveyed methods (N=100). Bold statistics in the table represent the highest performer for each metric and species. Precision values for *P. bethaniensis* and *P. forresti* are low because these two species represent the classes with the fewest number of samples, and so there is a very small amount of true positive values. However, these still greatly exceed what would be expected by chance (0.001 and 0.006, respectively). The high recall for these classes indicates that the true positives are correctly captured.

### Characterization

The data acquisition procedure is not without noise, so we perform filtering to produce accurate quantitative characterization of flash phrases that falls in alignment with previous literature observations. We leveraged the ability of the RNN to distinguish between sequences by choosing the sequences which the RNN scored highest as the top one hundred most confident classifications for each species. This subset acts as the dataset on which characterization exercises are performed for Fig. 1B and Fig. 4. The procedure is as follows:

1. Initialize the empty list *c*
2. for each *i* sequence in the test set *D*:
  - Run a forward model step
  - Let *p* = the maximum probability in the resulting vector of softmax predictions
  - If the index of *p* corresponds with the correct label, add the pair (*p*, index) to the list *c*
3. Sort *c* by probability *p* and choose the top 100
4. Index into the dataset D using the associated indices of the top 100 probabilities to produce the subset

Characterizing in this way leverages the variability in the entire dataset by training the predictive classifier, then asks the predictive classifier only for what it is most confident about in order to filter out sequences that may be missing flashes or exhibiting patterns that are far from the statistical norms of the species.

### Supplementary Information

Supplementary Information.

## Data Availability

The associated code and data files for this project can be found here: https://github.com/peleg-lab/FireflyClassification. Following instructions in the associated README.md should allow easy regeneration of the figures and replication of the workflows described in this paper. All data needed to evaluate the conclusions in the paper are present in the github repository, the Supplementary Information, and/or the associated dataset paper^24^.
