# Supplementary material for: Embracing firefly flash pattern variability with data-driven species classification
Source: Sci Rep. 2024 Feb 10;14:3432. doi: 10.1038/s41598-024-53671-3 (PMC10858911; doi:10.1038/s41598-024-53671-3)
Supplement: Supplementary file 1 — Supplementary Information. [file 41598_2024_53671_MOESM1_ESM.pdf]

Supplementary Information

5.4 Batch effects

The dataset was assembled by several scientists operating at different locations across different years, so it is important to consider the possibility that the network is isolating a batch-related feature. We control for this in several ways: first, the methodology for video collection is standardized following procedural guidelines outlined in [24]. These are easy to follow, clearly delineated, and often quality checked before the data are gathered. Second, the videos are post-processed to transform them into binary sequences, eliminating extraneous factors that may normally contribute to batch effects in neural network training. Third, the resulting sequences are all transformed to the same time scale to avoid preference given to a certain frame rate. We believe these steps are sufficient to remove any noticeable effect generated by minor differences in data collection moving forward, but testing this is important to make sure the model is not learning a characteristic of the sequences that is isolated to a certain data gatherering event. To test this, we performed 5-fold cross-validation on the recurrent neural network where all of the data for a particular data gatherer was held out of the training set. For each fold, we isolated all of the *P. frontalis* and *P. carolinus* sequences in the dataset coming from R.S. [24]. We did this because *P. carolinus* and *P. frontalis* are two of the species for which there are multiple data-gathering events and camera operators present in the dataset, and R.S. took part in some of the data collection for each of these species. Holding their data out from the training dataset means the model will not be able to see any of those sequences, so if there is a batch effect, it should not be able to recognize them. Then for each of the 5 folds, we selected a different fold of the remaining *P. carolinus* and *P. frontalis* data to be part of the training set. The other species were treated normally, and the results are in Fig. S1. It is clear that there is no batch effect, as the model performs just as well during the 5-fold cross validation as it does without any data holdout. This is likely thanks to the distinctive flash duration of *P. frontalis*; additionally, despite the differences *between* distributions of the inter-flash gap for *P. frontalis*, the overall distribution is still uniquely situated between 0.5s-0.8s, which is unlike any of the other species in the dataset. This analysis supports the ability for our model to scale with different volunteers acting in different locations serving as the data-gatherers, and eventually to automated camera setups, without concern.

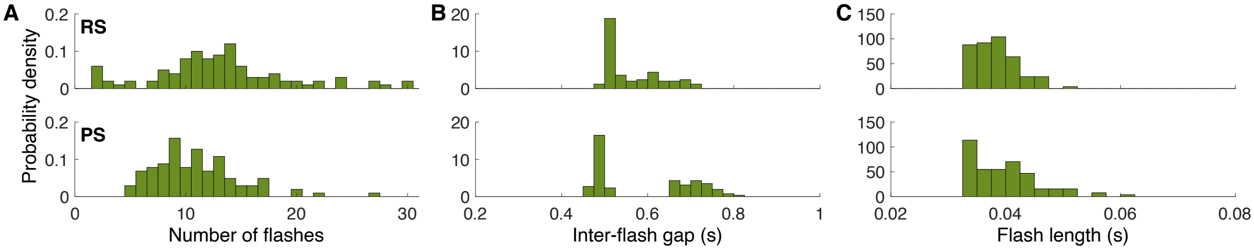

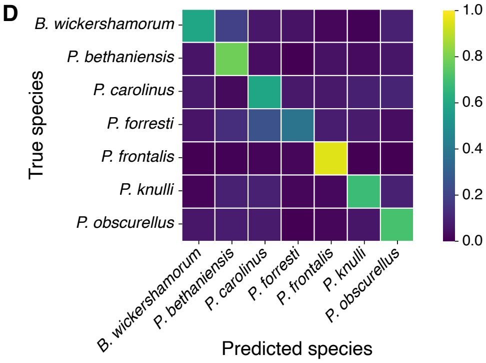

E

Species	Precision	Recall
B. wickershamorum	0.65	0.59
P. bethaniensis	0.15	0.78
P. carolinus	0.94	0.59
P. forresti	0.034	0.38
P. frontalis	0.74	0.95
P. knulli	0.40	0.68
P. obscurellus	0.27	0.71

Figure S1: Quantification of batch effects. **A-C:** Probability distributions of flash parameters from *P. frontalis* sequences collected by two different data gatherers, labeled RS (top) and PS (bottom). The distributions for number of flashes **(A)** and flash duration **(C)** are similar, while the inter-flash gap **(B)** distributions are notably different due to different temperatures during data collection for the two different gatherers. **D:** Confusion matrix for 5-fold cross validation of RNN where all *P. frontalis* and *P. carolinus* data gathered by R.S. is held out from training. The RNN achieves similar performance to the model described in the main text, which was trained on both RS- and PS- collected *P. frontalis* data. **E:** Table of per-species precision and recall during batch effect cross-validation.

5.5 Literature ROC results

The literature methods perform poorly compared to population reference methods, with dynamic time warping achieving the best weighted precision (0.77) but low weighted recall (0.12), and dot product achieving lower precision (0.57) but the best recall of all methods (0.41). The receiver-operating characteristic (ROC) curves for the literature-based methods are shown in Fig. SI2, along with the per-species precision and recall. All four methods perform poorly on *P. forresti*. SVM and DTW result in high recall on *P. bethaniensis*, but both classifiers tend to incorrectly predict many other sequences as belonging to this species. All methods achieve good precision on *P. carolinus*, but most demonstrate poor recall.

These results reveal that the existing literature-based reference patterns fall short in accurately representing our collected dataset. This may be due to the different conditions under which the literature-based references; moreover, by creating population references, we can better capture the behavioral variability found in the data.

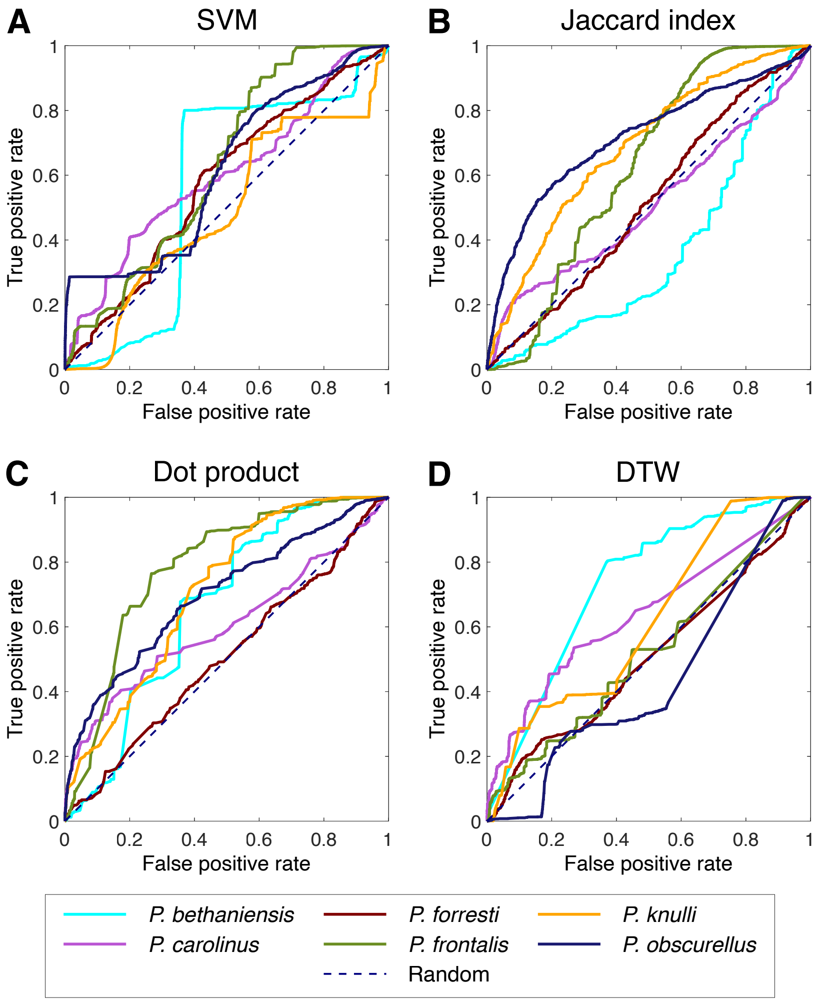

E

	SVM		Jaccard		Dot product		DTW	
Species	Precision	Recall	Precision	Recall	Precision	Recall	Precision	Recall
P. bethan.	0.017	**0.82**	0.0042	0.069	0.011	0.13	**0.022**	0.80
P. carolinus	0.83	0.082	0.72	0.23	0.73	**0.54**	**0.96**	0.093
P. forresti	0.012	**0.43**	0.0099	0.095	0.013	0.13	**0.016**	0.14
P. frontalis	0.27	0.036	0.34	**0.43**	0.31	0.028	**0.34**	0.094
P. knulli	**0.44**	0.33	0.14	**0.40**	0.099	0.29	0.11	0.39
P. obscur.	**0.45**	0.019	0.12	0.23	0.25	**0.25**	0.0039	0.013

Figure S2: Classification results for literature-based references. A-D: Receiver operating characteristic (ROC) curves representing the true positive rate (TPR) as a function of the false positive rate (FPR) across all model thresholds of classification, labeled by method. E: Table of per-species precision and recall.
